# Knowledge of family health program practitioners in Brazil about sickle cell disease: a descriptive, cross-sectional study

**DOI:** 10.1186/1471-2296-12-89

**Published:** 2011-08-19

**Authors:** Ludmila MX Gomes, Magda M Vieira, Tatiana C Reis, Thiago LA Barbosa, Antônio P Caldeira

**Affiliations:** 1 Department of Nursing, State University of Montes Claros, Montes Claros, Minas Gerais, Brazil; 2 Municipal Health Department, Januaria, Minas Gerais, Brazil; 3 Department of Women's and Children's Health, State University of Montes Claros, Montes Claros, Minas Gerais, Brazil

**Keywords:** Sickle cell anemia, Child, Quality of health care, Primary health care

## Abstract

**Background:**

Although sickle cell disease is an important public health problem in Brazil, there is a gap in the literature on the level of knowledge of primary health care professionals about the treatment and management of sickle cell disease. Therefore, this study aimed to evaluate the level of knowledge about sickle cell disease of physicians and nurses who work in the Family Health Program in a region of Brazil with a high prevalence of this disease.

**Methods:**

This is a descriptive, cross-sectional study conducted at the municipality of Montes Claros, in the north of Minas Gerais, Brazil. Study participants included 96 physicians and nurses who work at the Family Health Program in an urban area of the city. Data was collected using an original, partially tested questionnaire based on health care check points for children with sickle cell disease established in educational protocols from the State Health Secretary of Minas Gerais and the Ministry of Health. The structured questionnaire contained 47 questions addressing three axes: epidemiology (8 questions); clinical manifestations (13 questions); and management of children with sickle cell disease (26 questions). Knowledge was measured through mean correct responses to proposed questions. Ethical principles were respected and this project was approved by the Committee of Ethics in Research.

**Results:**

59.4% (57) of the study participants were nurses and 40.6% (39) were physicians. The median length of training and median length of service in primary health care were 4.3 (2.8-8.0) years and 4.0 (2.0-7.1) years, respectively. The mean performance in knowledge tests was < 75%, with 5.7/8 (SD = 1.4) for the "epidemiology" questions; 8.6/13 (SD = 2.2) for "clinical manifestations"; and 17.0/26 (SD = 2.9) for "management of children with sickle cell disease" questions; resulting in a mean total of 31.4/47 (SD = 5.10) correct responses. A statistically significant association was found between the number of correct responses and family health care qualifications (p = 0.015).

**Conclusion:**

There is an urgent need to improve primary health care professional training in the care of children with sickle cell disease.

## Background

An estimated 7% of the world population is affected by hemoglobin disorders, represented mostly by thalassemia and sickle cell disease. The latter, due to a genetic change in hemoglobin, is the most common hereditary hematologic disorder in Brazil and the world [1]. In Brazil, the prevalence of sickle cell anemia is estimated between 25,000 and 30,000 cases [2]. One characteristic of sickle cell disease is its clinical variability. Some patients develop a more severe variant of sickle cell disease and suffer from additional complications and frequent hospitalizations, while others present with more benign symptoms, or are even asymptomatic. Although both inherited and acquired factors contribute to this clinical variability, socio-economic status stands out as one of the main factors affecting clinical manifestations, implying the importance of changes in food quality, the potential for the prevention of infection and timely access to quality health care services [3-5].

In addition, clinical manifestations of sickle cell disease vary over time, ranging from periods of wellness to the need for emergency care, suggesting hierarchical levels of disease complexity which require equally complex health care [3]. Unfortunately, studies have shown that many professionals in primary health care are unaware of, or even ignore, sickle cell disease [6,7].

Some authors emphasize that awareness of sickle cell disease, a chronic illness which has an enormous impact on entire families, and health care training in its treatment, should be widely implemented in many health care services [6-8]. Even in countries where there is a low prevalence of sickle cell disease, some authors suggest the need for training health care professionals in primary care of this disease [9]. In Brazil, we have some areas with high prevalence of the disease. In such areas children with sickle cell disease are more vulnerable than adults. In this context, it is necessary to investigate whether doctors and nurses who serve in these areas are adequately trained and prepared to assist these children and their families.

In the last few decades, Brazil has implemented important health care reforms, with the deployment of multidisciplinary teams for decentralized primary health care (known as the Family Health Program). Despite some positive results from these reforms [10], to date, no studies have attempted to measure the level of knowledge of these health care professionals regarding the management of diseases that depend on enhanced primary care. It is questionable if these primary health care professionals, whose original training is based on a curative and hospital model, are prepared to meet and properly manage patients with sickle cell disease, anticipate risk situations, and guide and lead the patient's family during periods when the disease manifests with greater severity.

Considering recent advances in the treatment of children with sickle cell disease, it is increasingly important to ensure the adoption of effective practices and integrate them into a context that meets the needs of afflicted children and their families [11]. Unfortunately, primary care professionals working in environments remote from major centers are, in general, not fully aware of guidelines for the treatment and management of these patients [7]. For example, studies in countries such as Ghana and Great Britain have uncovered poor performance by health professionals in relation to the treatment and management of sickle cell disease and current medical education programs were considered to be insufficient for the development of skills to care for patients with this disease [12,13].

Because of its prevalence and wide-spread consequences, sickle cell disease is an important public health problem in Brazil. In spite of this, there are gaps in the literature on the level of knowledge of primary care professionals about the treatment and management of this disease. Considering the importance of the primary care system in the current Brazilian health care system, an understanding of these issues is highly relevant to public health policies. Therefore, the aim of this study was to assess the knowledge of medical professionals and nurses working in the Family Health Program in Brazil about sickle cell disease in an area of high disease prevalence.

## Methods

### Study design

This is a descriptive and cross-sectional study conducted in the municipality of Montes Claros. Montes Claros is the principal urban center of the region and is located north of Minas Gerais, Brazil. With a population of approximately 360,000 residents, Montes Claros has 52 teams from the Family Health Program working in the urban area and 7 teams working in rural areas.

### Participants

All physicians and nurses working in family health teams in the Montes Claros urban area were eligible to participate in this study. Study exclusion criterion included being on vacation or work leave at the time of data collection.

An original, partially tested questionnaire was developed based on the most important aspects of caring for children with sickle cell disease, as established by the policies of the State of Minas Gerais and the Ministry of Health [14,15]. The questionnaire was assessed for content validity by five medical practitioners, including two specialists, two pediatricians and a nurse with expertise in primary care. The questionnaire was evaluated for the presence or absence of the following criteria: comprehensiveness, objectivity and relevance. A pilot study was conducted on professional teams working in family health in rural areas. The professionals of these areas did not participate in the study.

### Variables

The study participants were characterized using the following variables: gender (male/female); age (years); marital status (married or stable relationship/single, separated or widowed); professional category (nurse/physician); elapsed time since graduation (years); length of service in Primary Care (years); specialization (qualification) in family health (yes/no); and knowledge of the presence of sickle cell patients in the geographic area of the Familiy Health Program (yes/no). In this study, a specialization (qualification) in family health was defined as a residency (medical or multi-professional) in family health lasting at least two years, coupled with a full course load (60 hours per week) covering both theoretical and practical aspects of family health.

The level of knowledge of family health care workers about sickle cell disease was assessed by their answers to true or false statements in the questionnaire. The questionnaire contained 47 questions divided into three sections: Epidemiology (8 questions); Clinical Manifestations (13 questions that addressed pathophysiology, pain crises, acute splenic sequestration, acute chest syndrome, stroke and infections); and Management of children with sickle cell disease (26 questions about the care environment, hydration, growth and development, nutrition, vaccination, prophylactic antibiotics, use of folic acid, physical examinations, warning signs, specialty visits, transfusions, genetic counseling, and guidance to parents among others).

### Analysis

The knowledge test performance was measured using the average number of correct answers to the questions. To simplify assessment of the study participants' results, total scores were categorized as follows: below average performance (< 31 hits) and above-average performance (≥ 31 hits). The chi-square test with Yates correction was used to examine the correlation between the dependent variable (performance below/above average) and other variables. Variables in which differences were statistically significant for the overall score of the study participants were sought to assess more accurately the average scores in different test areas. Student's T-test was used to compare mean values. Verification of the normal distribution of values was performed using a Kolmogorov-Smirnov test.

### Ethical approval

In this study, ethical rules were respected and study participants signed a consent form. The project was approved by the Committee for Research Ethics of State University of Montes Claros with Opinion-No 1517/2009.

## Results

104 physicians and nurses from 52 family health teams were eligible and were invited to participate in this study. Of these, 96 participated in the study participation rate 92.3%: 59.4% (57) nurses and 40.6% (39) physicians. The median length of training and years of service in Primary Health Care were 4.3 (2.75 to 8.00) and 4.0 (2.00 to 7.06), respectively. Most of the study participants were female (70.8%), with a median age of 29 years (22-59 years). The marital status of the study participants was predominantly listed as stable or married (54.2%) and most respondents had no children (58.3%). The main characteristics of the study group are presented in Table 1. Regarding general awareness of local sickle cell disease, 45.8% of the study participants were aware that sickle cell patients were in the geographic area.

**Table 1:** Characteristics of study participants, Montes Claros, Minas Gerais, Brazil, 2010

Variable	N	%
Sex		
Male	28	29.2
Female	68	70.8
Number of children		
None	56	58.3
1-2	35	36.5
3 or more	5	5.2
Marital status		
Married or stable relationship	52	54.2
Single or divorced or widower	44	45.8
Education		
Nurse	57	59.4
Physician	39	40.6
Qualification in Primary Health		
Yes	60	62.5
No	36	37.5
Time since graduation		
≤ 5 years	54	56.3
> 5 years	42	43.7
Length of service in Primary Care		
≤3 years	43	44.8
4 to 7 years	30	31.3
≤ 8 years	23	23.9

The average performance of the study participants on the sickle cell disease knowledge test was < 75% in all test areas. The description of the medium is shown in Table 2. Table 3 shows the results of primary health care worker performance. There was a statistically significant correlation between study participants' answers to the question "Are there patients with sickle cell disease in the family health care operational area? (Yes/No)" (p = 0.015) and overall test performance.

**Table 2:** Scores and percentages relative to the performance of professionals in the test of knowledge about sickle cell disease

Section	Number of questions	Scores and percentages		
		
		Minimum	Maximum	Mean
		n (%)	n (%)	n (%)
Epidemiology	8	3 (37.5)	8 (100.0)	5.7 (71.6)
Clinical Manifestations	13	3 (23.0)	13 (100.0)	8.6 (66.6)
Management of children with sickle cell disease	26	8 (33.8)	22 (84.6)	17.0 (65.5)
Total correct	47	19 (40.4)	41 (87.23)	31.4 (66.9)

**Table 3:** Association between variables and performance on the tests of knowledge about sickle cell disease among professionals working in the Family Health Program; Montes Claros, Minas Gerais, Brazil; 2010

Variables	Performance below the average (<31) n (%)	Performance above the average (≥31)n (%)	p-value*	OR (95%CI)
Sex			0,679	
Female	22 (66,7)	46 (76,0)		0,74 (0,27-2,03)
Male	11 (33,3)	17 (27,0)		1,0
Children			0,913	
Yes	19 (57,6)	37 (58,7)		0,95 (0,37-2,44)
No	14 (42,4)	26 (41,3)		1,0
Marital Status			0,872	
Married/stable relationship	17 (51,5)	35 (55.6)		0,85 (0,34-2,15)
Single/divorced/widower	16 (48,5)	28 (44,4)		1,0
Education			0,967	
Physician	14 (42,4)	25 (39,7)		1,12 (0,44-2,87)
Nurse	19 (57,6)	38 (60,3)		1,0
Time since graduation			0,685	
≤ 5 years	20 (60,6)	34 (54,0)		1,31 (0,51-3,38)
> 5 years	13 (39,4)	29 (46,0)		1,0
Length of service in Primary care			0,838	
< 3 years	25 (75,8)	48 (76,2)		0,98 (0,33-2,93)
≥ 3 years	8 (24,2)	15 (23,8)		1,0
Qualification in Family Health program			0,067	
No	17 (51,5)	19 (30,2)		2,46 (0,95-6,45)
Yes	16 (48,5)	44 (69,8)		1,0
Are there patients with sickle cell disease in the operational area?			0,015	
No	24 (72,7)	28 (44,4)		3,33 (1,23-9,23)
Yes	9 (27,3)	35 (55,6)		1,0

* Pearson Chi-square test with Yates correction.

Table 4 shows an analysis of the mean scores from different sections of the test. Statistically significant differences were observed for all sections when study participants' responses were sorted according to their response to the question, "Are there patients with sickle cell disease in the family health care operational area (Yes/No)?".

**Table 4:** Average performance of professionals working in family health care in tests of knowledge about sickle cell disease, sorted according to whether they were aware of the presence of patients with sickle cell disease in their operational area according to the sections studied, Montes Claros, Minas Gerais, Brazil, 2010

Section	Are there patients with sickle cell disease in your operational area?	
	
	Yes	No
Epidemiology		
Mean	6.11	5.40
Standard Deviation	1.29	1.47
p-value*	0.015	
Clinical Manifestations		
Mean	9.23	8.17
Standard Deviation	1.71	2.34
p-value*	0.015	
Management of children with sickle cell disease		
Mean	17.68	16.50
Standard Deviation	2.10	3.00
p-value*	0.031	
Total hits		
Mean	33.05	30.08
Standard Deviation	3.41	5.38
p-value*	0.002	

* Student's T-test. The normal distribution of values was verified by the Kolmogorov Smirnov test.

## Discussion

Indirectly, the level of knowledge measured reflects the quality of health care that doctors and nurses are providing to patients under their responsibility. It is disheartening that all observed scores were below 75%, indicating a level of knowledge below desired levels. Furthermore, the questions asked in this study were based on guidelines set by the Ministry of Health and the State Health Department, implying that participating health care professionals were unaware of such guidelines.

The overall performance of the study participants on the "Epidemiology" test section was less than desirable. Knowledge of the epidemiology of a disease is important, because it enables an understanding of the distribution and determinants of health and disease processes in human populations. In particular, knowledge of sickle cell disease epidemiology would enable the health care professionals participating in this study to understand the magnitude of the disease in their region and alert them to factors associated with negative outcomes of the disease. Moreover, a thorough knowledge of disease epidemiology would enable the development of group or risk stratification for patients and families under their responsibility. This study assessed the level of knowledge of physicians and nurses about sickle cell disease in the northern region of Minas Gerais, an area of Brazil with a high prevalence of the disease. This Brazilian state has the highest incidence of disease as measured by newborn screening [2].

For the "Clinical manifestations" section, scores varied from 23.0% to 100.0%, with an average score of 66.6%. Sickle cell disease affects many aspects of clinical medicine (social, clinical, hematological, genetic, biochemical, etc.) due to its high morbidity and high mortality rate. The most common clinical problems are painful crises, along with other relevant complications, such as acute splenic sequestration, acute chest syndrome, bacterial infections and stroke. Doctors and nurses should have knowledge of these clinical manifestations of sickle cell disease, which represent an essential guide to the direct patient treatment [6,16].

The low performance of study participants on the test section dealing with the "treatment of infants with sickle cell disease" indirectly implies the existence of significant problems in family health care. It is striking that health care professionals participating in this study do not have the necessary knowledge about the specifics of treating infants with sickle cell disease with respect to growth and development, physical examinations, special vaccines, the use of prophylactic penicillin and folic acid, laboratory tests, specific guidelines for families, and referrals to dentists or opticians. Because sickle cell disease is a chronic condition that usually brings with it a high degree of suffering, especially in children, sickle cell disease deserves special attention. Importantly, the medical and psychosocial aspects of sickle cell disease should be well known by primary health care providers [6,16].

Doctors and nurses associated with primary care services have a commitment to longitudinal patient care, and knowledge of the aspects evaluated in the present study are essential for planning patient care. In addition, scientific knowledge provides a basic foundation for the work of health care professionals. Thus, it is expected that individual consultations and targeted home visits, effective actions to control painful crises and other signs or symptoms, educational and genetic counseling, prevention of infection, monitoring of antibiotic prophylaxis, vaccination and screening for the risk of stroke, which are essential aspects of the treatment and management of sickle cell disease, are best performed by knowledgeable health care professionals [17].

The quality improvement, highlighted as an indispensable part of health care, is not the reality for sickle cell disease yet [7]. Although there have been few studies about the level of knowledge and performance of health care professionals in relation to sickle cell disease, the poor results observed in this study do not appear to be unique. For example, in England, a 2008 report of the National Confidential Enquiry highlighted a lack of knowledge of physicians and nurses in the management of patients with sickle cell disease in the hospital environment [18]. A previous study in the same country found that the training received and the skills developed in nursing education are insufficient for the management of patients with sickle cell disease. Furthermore, approximately 60% of nurses felt unprepared to deal with these patients. In fact, when tested on their knowledge of the treatment and management of sickle cell disease, the average performance of these nurses was only 31%, below the results found in this study [13].

Knowledge gaps related to the treatment of sickle cell disease were also identified in a survey conducted to assess the educational needs of health care workers and patients in Ghana, before the introduction of neonatal screening for sickle cell disease. In the study, 22 doctors and 35 nurses from clinical care services were interviewed [12]. The results demonstrated the need for organized continuing education for doctors and nurses to provide qualified assistance and know-how to manage the neonatal screening program [19]. Similar results have been identified in others countries: In Nigeria a study carried out among health professionals and medical students showed for example that only 55% of the respondent felt genotype screening should be done at pre-school age and only 24.3% knew most of the complications of sickle cell anaemia. The authors conclude that continuing medical education for health professionals about sickle cell anaemia, its management and complication is necessary [20]. Others studies have assessed the knowledge of sickle cell disease in some communities and all of them conclude that is there is need to sensitise communities and policy makers about the disease, including its screening and adequate management [21-23].

In Brazil, some authors [6,8,24] have pointed out the need for training primary care professionals to provide better care for patients with sickle cell disease. In the process of reshaping health care in Brazil to place a greater emphasis on family health, it is important that family health care teams are well-prepared to adequately serve patients with sickle cell disease. Disease monitoring by the Family Health care team is a valid strategy for improving the level of medical attention received by this group of patients, who suffer from both environmental and intrinsic factors associated with this chronic disease.

In this study, knowledge of the presence of patients with sickle cell disease in the geographical area covered by the family health unit (OR = 3.33, 95%CI 1.23 to 9.23) was correlated with better scores of health care professionals. The authors believe that the presence of the field unit induces the patient to search for professional expertise, and for updates about the disease. These results point to the importance of the attributes of primary care, especially longitudinal care, centralization, and coordination of care family. Longitudinal care is the contribution of a regular health care team and its consistent use over time, providing a humane environment for mutual relationships between the health care staff, patients and their families. Coordination of care is characterized by ensuring continuity of care within the service network, thus enabling comprehensive care. Centralization of family health care suggests that the family is the subject of medical attention, implying interaction with a healthcare team that has full knowledge of their health problems [25]. In addition, the ongoing care of patients with sickle cell disease and their families also implies a hierarchy of assistance with referrals to specialist consultations and the involvement of other levels of health care. It is believed that all of these principles can intuitively be present in the care of children with sickle cell disease in the Family Health Program, which explains the better scores of professionals who were aware of patients with sickle cell disease on the knowledge tests.

### Limitations of the Study

Naturally, there are some limitations to the generalability of these findings, because the present study was confined to a municipality that has the distinction of being the residence seat of medical and multi-professional primary healthcare. However, the lack of national and international studies paves the way for other studies assessing the level of knowledge of these health care professionals. Another limitation refers to the data collection instrument used: an original questionnaire partially only tested. It is worth noting that there are no validated instruments to assess the level of knowledge of healthcare professionals on this subject. This study was not designed to obtain data about the actual practice of the healthcare workers, but only their level of knowledge about sickle cell disease in children. In addition, it must be highlighted that due to the small sample size analysis was not performed for each professional category separately (physicians vs. nurses).

## Conclusion

This study describes the level of knowledge of primary health care workers in Brazil about sickle cell disease. In addition, this research points to the necessity for training these professionals, because their performance on knowledge test was inadequate and insufficient can indirectly interfere with their ability to provide appropriate health care.

Awareness of the presence of sickle cell patients in the geographical area of operation positively correlated with the level of knowledge of the primary care professionals about sickle cell disease. However, the level of knowledge found in this study is still unsatisfactory for the treatment and management of children with sickle cell disease. Thus, there is an urgent need that there is an urgent need for training primary care professionals to improve health care quality for children with sickle cell disease and for securing the dignity and citizenship rights of such patients. It is necessary to conduct continuing education for primary healthcare professionals to improve their ability to care for, and provide guidance to, patients with sickle cell disease and their families.

We think that continuing education of health care professionals (in particular physicians and nurses of primary health care teams) on management of patients with sickle cell disease is necessary to ensure better quality in health care. This continuing education must be based in real situations and professionals should be encouraged to act as multipliers of knowledge acquired. Primary health care teams should be stimulated to discuss with professionals from hospitals and blood centers the best way to care for their patients with sickle cell disease. So they will actually build an effective network of health care. Further studies, testing the knowledge of healthcare professionals before and after training in the treatment and management of sickle cell disease would allow continued discussion of this topic.

## Competing interests

The authors declare that they have no competing interests.

## Authors' contributions

LMXG and APC participated in the design, project coordination, data analysis and drafting of this article. MMV, TCR, and TLAB participated in data collection, data analysis and drafting of this article. All authors read and approved the final manuscript.

## Pre-publication history

The pre-publication history for this paper can be accessed here:

http://www.biomedcentral.com/1471-2296/12/89/prepub
